# Methodological challenges in outcomes research for early-trials for implementation of new therapies in neuropediatric rare diseases

**DOI:** 10.1186/s13023-025-03814-0

**Published:** 2025-10-17

**Authors:** Maria T. Acosta, Silvia Zaragoza Domingo, Celso Arango, Kim I. Bishop, Joan Busner, Georg Dorffner, Inés del Cerro, John Carlos Diaz, Edna Gruenblatt, Sabine M. Hölter, Brian Harel, Vanitha Krishna, Marta Mas, Carmen Moreno, Stefano Pallanti, Carme Plasencia, Sarah Kittel-Schneider, Daniella Tinoco, Monica Vance, Manpreet K. Singh, Simona Giorgi, José Ángel Aibar, Antonella Santuccione Chadha, Estibaliz Arce Cirauqui

**Affiliations:** 1 NHGRI: Undiagnosed Disease Program, National Human Genome Research Institute, NIH, Bethesda, USA; 2 Neuropsychological Research Organization s.l (Neuropsynchro), Barcelona, Spain; 3 Hospital General Universitario Gregorio Marañón, Madrid, Spain; 4 Global Pharma Consulting Ltd, Aylesbury, USA; 5 Signant Health, Blue Bell, USA; 6 The Siesta Group, Vienna, Austria; 7 UCAM: Universidad Catolica San Antonio de Murcia, Murcia, Spain; 8 Geosera Ltd, Berwyn, USA; 9 University of Zurich, Zurich, Switzerland; 10 Technical University of Munich, Munich, Germany; 11 Takeda Pharmaceuticals America Inc, Cambridge, USA; 12 Barcelona, Spain; 13 Instituto di Neuroscienze, Pisa, Italy; 14 Aromics, Barcelona, Spain; 15 Cork University Hospital, Cork, Ireland; 16 Worldwide Clinical Trials, Barcelona, Spain; 17 Santium, Toronto, Canada; 18 University of California Davis, Davis, USA; 19 Dravet Foundation, Madrid, Spain; 20 Dravet Foundation, Nice, Paris, France; 21 Women`s Brain Project, Zurich, Switzerland; 22 Engrail Therapeutics, Inc., San Diego, USA

## Abstract

During 2023, the leads of the ECNP TWG focused on Clinical Outcomes in Early-Trials in Neurosciences scheduled two brainstorming sessions to allow a deep discussion about challenges facing neurosciences drug development in neuropediatric rare diseases. Sessions were led by Dr Maria T. Acosta from the Undiagnosed Disease Program (UDP) at the National Human Genome Research Institute, National Institutes of Health, Bethesda, Maryland, EE.UU. The experts discussed about challenges and potential solutions as well as alternative options to design appropriated for clinical outcomes assessment (COA) instruments for this population. An important discussion took place due to extensive expertise of the participants and hands-on experience with clinical trials. Participation from experts from several disciplines was a key factor for appraising and brainstorm innovative solutions in the field. Identification of challenges and propose of innovative solutions were a central core of the discussion. Despite of the increasing number of rare and ultra-rare diseases being identify by the day, and the significant differences in biology, and clinical presentation, it is clear most of them face similar problems when is time to select the appropriated COAs to test potential interventions, and experts have a limited potential to drive efficient solutions. Some of the commonly identified common problems include: small number of patients affected, disease changes over time, developmental aspects impacting the clinical presentation according with age, individual variability in terms of disease severity between patient, variable window for intervention and in some cases, need to expedite treatment as per the disease progression. All these and other features, require an extensive dialogue and continue communication between preclinical researchers, clinicians, patients and family members, pharma and treatment designers and regulatory agencies in each condition, making this process, expensive, time consuming and very difficult to accomplish. A feasible solution, that may be applicable to several conditions, is to develop a “back bone” structure to approach rare diseases, allowing each “disease team” to tailored assessments and study design, according with the specific features of the condition. We concluded that it is fundamental to establish effective bridges of communication between the different actors implicated in the clinical trial design and execution, sharing experiences, as well as clear understanding of meaningful outcomes not only for researchers, but clinicians, patients and families. Important emphasis is done in the need or careful selection of COAs in each individual condition to be able to obtain more efficient and reliable results in clinical trials in this population. We need to be creative, and there is a need to leave the “comfort zone” of current methodologies and start piloting new methods at clinical settings. A specific research platform was proposed as a solution in the creation, sharing and validation of new assessment instruments, which would be available to clinicians and researchers attending small patient samples distributed over the world.

**Letter to editor**.

Recent advances in basic and clinical research as well as therapeutic science are producing increased opportunities for clinical trials in a wide variety of medical diagnoses. As those advancements are being applied to a variety of medical conditions, patient ages, and levels of cognitive compromise, children affected with rare diseases merit special attention, as many of them also present with intellectual and developmental disabilities. This cohort of youth is especially challenging to study in the context of a clinical trial due to variability in age, severity, and developmental status at the time of screening.

The United States (US) Food and Drug Administration (USFDA), and consistent with the Orphan Drug Act, defines a rare disease as one that affects fewer than 200,000 people in the US [2, 13]. Around 50% of rare diseases affect children and in the majority are characterised by neurodevelopmental alterations and intellectual disability [2, 9, 13]. Furthermore, in patients with severe intellectual disabilities there is a higher chance of a significant genetic contribution to their condition than in those without severe intellectual disabilities [2, 9].

Important advances have been made lately in terms of novel experimental therapies and study designs for these conditions. For example, several clinical trials have started to treat youth with Fragile X, Angelman syndrome and Rett syndrome for targeted interventions [1, 3, 6, 10, 18, 19], yet only some positive results for Rett syndrome have been reported. Similarly, step forward efforts have been made in many other conditions with like Duchenne muscular dystrophy (DMD), Spinal Muscular Atrophy (SMA), for which a disease modifying treatment was approved however, despite the important results, there is room for improvements for patients to come.

However, one of the most significant challenges in conducting these trials relates to outcome selection, or our ability to demonstrate an objective and measurable impact of the intervention with well-grounded methodology. Just as with any clinical outcome assessment (COA), in a clinical trial setting, tools to assess efficacy should measure concepts that (1) are clinically meaningful to patients, caregivers, and clinicians and (2) possess robust psychometric properties, i.e., high validity, reliability and responsivity to change [7, 8]. In attempting to move forward with endpoint/outcome selection, more questions than answers arise. For example, how is a clinically meaningful change in outcome defined, how are treatment-related changes measured, how is selection of a COA validated, or proved to be reliable, or responsive to change? There may be inherent challenges to address these questions, especially as responses may be limited by key characteristics of these rare conditions: e.g. scarce data on natural history of the disorder, small sample sizes, heterogeneity in severity of presentation, course and outcome of the disease, overlapping biological markers, and limited information from natural history studies.

A recently created experts’ group on Outcomes Research in Neurosciences met virtually in May 2023, to discuss the challenges observed during the participation in clinical trials for rare neuropediatric indications. The initial discussion started commenting on recent clinical trials as example cases which evolved into a broader discussion about challenges designing and conducting research of novel therapies for rare diseases.

During the discussion, the authors advocated for the inclusion of several potential clinical outcome strategies that might prove more efficient than those currently in place which present with several limitations. Experts were members of the recently created thematic working group (TWG) Outcomes Research in Early-Trials as well as members of other constituted TWG and networks within the European College of Neuropsychopharmacology (ECNP). Attendees represented different stakeholders including, clinicians, psychiatrists, neurologists and researchers in the pharmaceutical industry, biotech, academia, and service-provider companies for clinical research.

Many of the experts were also participants in the ongoing International Society of CNS Clinical Trials Methodology (ISCTM) Orphan Diseases Working Group. The Orphan Diseases Working Group has published several related papers directed to CNS rare disease drug development [5, 17] as well as pediatric CNS drug development [4, 11, 16].

Through collective appraisal, suggestions were made to advance outcome strategies in neuropediatric rare disease research targeted. Advances in outcomes selection and outcomes generation, will help to provide reliable research to explore therapeutic interventions to eventually improve functioning and quality of life among patients and families suffering from rare diseases. Several notable areas of improvement are highlighted below and in Table 1.

There was consensus that many trials in neuropediatric rare diseases are hindered by inefficiencies in outcome measures available and selected for those trials. This has motivated the writing of this letter in which we express concern about current practices in neuropediatric rare diseases clinical trials. Highlights of the concerns include:

- Need for more comprehensive, valid, reliable, and meaningful COAs instruments (OMIs) than the existing methodologies and currently available tools. Recognition of the limitations and special needs for this population is fundamental to advance this field which is different from other fields with larger patient samples.
- Lack of tailored designed methodology for small clinical trials. Rather we are using adaptations of traditional methodology for clinical trials with modifications according with the research team perception of needs, but without clear methodological support of those modifications. Consequently perpetuating methodological problems issues in study designs and lack of reproducibility.
- Weak/poor/lack of correlation between neurobiological knowledge and understanding and design and use of COA instruments. Lack of correlation of biomarkers with immediate clinical manifestations or changes. Short duration of the trials making impossible to drive biological conclusions over process that had taken very long time to produce measurable clinical manifestation.
- Need to include the caregiver voice in rare/ultra-rare conditions in children. Need for design valid measurements to capture subjective changes observed during follow up as by parents and caregivers living with the patient, i.e. observers reported outcomes (ObsROs). Methods linking objective measures to parent-observed or tutor-observer changes over time associated with the intervention may help bridge this gap of lack of sensitivity of currently used OMIs. Researchers working in CT in rare diseases, need a deep knowledge and education on developmental aspects in children and how they can impact the selection or results on selected COAs.

Methodological unmet needs in clinical trials, we present at Table 1 identified limitations mentioned in the context of clinical trials in neuropediatric rare diseases.

**Table 1:** Table with identified limitations mentioned in the context of clinical trials in neuropediatric rare diseases

Methodological challenges	Description of limitation	Alternative / cons
1. Reliability of clinical and biological outcomes.	• Measures are not 100% reliable as per biology of the disease.	• Look for innovative methods.
2. Standardized testing, psychometric issues (e.g., floor effects).	• Lack of sensitivity to detect change following interventions, due to limitations in current available test unable to assess complexity of patients in terms of variability in ages, severity of the disease among other variables. “One does not fit all”	• Wider available selection or creation of test to properly assess the real status of a patient. • Using multiple measurements at once (composite scores) to best assess “real life assessment”. • Using same patient as own control, or using natural history data as historical controls.
3. Compare profiles of rare diseases subpopulations to (with WNL) neurotypical groups.	• Lack of understanding impact of developmental trajectories and impact comparing neurotypical patients and affected patient with neurological conditions. • Specific disease related normative data requires large samples and can be a lengthy process, not available in rare diseases.	• Collaborative effort to as much as possible larger samples/ • Use of normative samples for standardization of results needs to be discussed beforehand to avoid losing sensitivity to detect change. • Options: comparing rare disease patients with matched neurotypical peer: how often a patient with the clinical conditions of interest differs from no affected peers (effect size) and whether these changes are reliable (i.e., significance) (Chelune, 2010; Smith).
4. Traditional neurological examination not sensitive enough to changes in CTs.	• Despite an apparent change, measuring is difficult due to several factors. Current measurements not sensitive enough.	• Same as row #2. • Use of “real life data” from parents or caregivers, with better day to day knowledge of the patient.
5. High carer expectations of the new treatments.	• This can have two types of effect; either observed improvement is considered too small or considered more than realistic (inflation of results by observers-“placebo effect”).	• Clear and written material from the beginning of the trail with explanations about expectations, unknowns, etc. • Include caregiver expectative Scores (Cg Exp) to use as a covariate of the endpoints evaluated by caregivers.
6. Patients/carers traveling long distances to the study site.	• Patients and family’s effort in traveling and time required for participation, impact short and long terms interactions. • Subjective information depends on person completing the form(s) and how well rapport is established, and instructions are given. As well as Patient’s performance and/or scores are dependent on the carer’s perspective/skills to establish a good rapport and generate a favorable/confident environment.	• Develop tailored methods for remote video-recording of patient for home evaluation. • Centralized rating with training and intermittent rater drift. • Need for development of clear protocols, and standard procedures and training for proper acquisition of video material.
7. Variability across clinical presentations of the same conditions.	• Difficulty to identify a clinical benefit on specific outcomes when there is heterogeneity of symptoms that can be present and with different severities.	• Explore alternative methods to personalize assessments patient by patient, including alignment with parents and use of patient as own historical control. • Explore approaches such as pharmacogenomics to minimize variability especially in in gene therapy trials.
8. Different age ranges of the participants in the study sample.	• Different time of disease evolution (from asymptomatic to severely impair). Changes overtime in assessment tools according with age. • Differences in cross sectional observation of disease severity according with patient age at time of enrollment.	• Consider specific inclusion/exclusion criteria in order to compensate for psychometric weaknesses of the tests.
9. Length of clinical trials tends to be too short to see improvements.	• Traditional clinical trials tend to have too short follow up periods, difficult to assess real impact of interventions over time, (developmental skills acquisition, and changes in slope of disease progression among other aspects).	• Use more ecological time frames based on natural development.
10. Interview-based assessments exploring adaptive behavior (e.g., Vineland Adaptive Behavior Scales-VABS [20].	• Interview based on subjective evaluations by observers of patient’s skills, not based on performance evaluations. Not possible to prove with objective data.	• Consider methods to corroborate the items of Vineland-3 in the context of clinical trials. For instance, create a Vineland-3 specific for CTs. • Combine data, use of subjective and objective data to validate results.
11. Comorbidities and treatment for comorbidities are a confusing factor.	• Measuring the impact of different treatments including rehabilitation, on the course and treatment	• Opportunity to develop how a follow up look like, and including the impact of treatments for different treatments. • Getting an agreement about which outcomes needs to be used at the clinical practice among professionals and with carers.
12. Lack of definition of functionality in the context of rare diseases.	• Some functional aspects in daily life are directly related to the quality of life of the patient.	• There is a need to provide a definition that can be suitable for rare diseases.
13. Outcomes based on Clinical Global Impressions (CGIs).	• Difficulties in use and concern about objective and reliable assessment of real problem and change.	• Separate disease sub-domains specific impression of change on severity, are becoming common as a useful adjunct to the one global impression of change by clinicians or patients/caregivers (CGI-C/PGIC/ CgGI-C). • Design and implementation of disease specific CGI measurement include patients and caregivers observations [12].
14. Acceptance of innovative outcomes by regulatory bodies.	• Some innovations can be difficult to be accepted by Food and Drug Administration (FDA)/European Medicines Agency (EMA).	• Better communication process, stablishing multidisciplinary consortiums, to properly discuss and brainstorm needs and anticipate potential set back before implementation and final study design. • Increase inclusion of representatives of regulatory agencies on initiatives (e.g., ISCTM Orphan Diseases Working Group) More is needed.
15. Variability due to multicentric trials with different raters evaluating clinical endpoints	• Variability on COA results related to different study sites and different raters conducting clinical evaluations.	• Plan for harmonization of the evaluation criteria of COA instruments across study sites. The implementation of central rating could be a difficult option in these indications (rater non-familiar to the patient), but other strategies should be implemented to increase the inter-rater reliability i.e., with certified training, data audits and regular calibration across raters.

WN Within Normal Limits, PerfO Performance-based Outcome,

## Main learnings– next steps

- Natural history databases can bring information about the natural evolution of the patients to be used as historical controls.

Considering all the ideas, an interesting topic is how to create individual trajectories for patients (one at the time) using different global impression measurements (clinical CGI, caregiver CgGI-S/C), or use of AI technology to specific features specific to the condition, or even goal attainment scaling (GAS) methods to personalize targeted improvement, among others.

To introduce the trajectory of the patient along the disease, recently Retrospective CGI of Severity/Change (R-CGI-S/C) has been developed to measure disease severity and change based on medical records [14, 15], with demonstrated utility be used to rate historical patients with adequate correlation with brain biomarkers.

- A feasible solution, applicable to a number of conditions, is to develop a general “back bone” to approach rare diseases facilitating to each team the creation of their own individual and tailored assessments i.e. OMIs following a standard methodology and undertaking collaborative validation research. Lessons learned so far can guide future innovative approaches. For instance, not all diseases have the same profile yet all face the same problems such as small numbers, disease changes over time, developmental aspects that impact the course and selection of outcomes, limited time to intervene, among others, rare diseases share similar challenges not resolved with the current outcomes in use.
- Therefore, there was consensus among the participants of an urgent need to build a specific research platform, proposed as a solution, for the creation, sharing and validation of new OMIs, which would be available for the pilot use on specific conditions of clinicians and researchers over the world.

## Conclusion

- Fifteen years of experience participating in clinical trials for rare diseases in neuropediatric conditions, give an advantage to guide future developments to increase the ***efficiency of clinical research***.
- Need a ***shift in mindset*** to break away from more traditional or familiar ways of thinking that might create barriers for advancing science and generate groundbreaking or paradigm-shifting “out of the box” ideas for solutions to these problems.
- May need to leave the “comfort zone” of current clinical trial methodologies and advance other trial design adaptations that facilitate learning. Alternative methods are currently being ***piloted as exploratory*** in clinical practice at clinical settings, and even if they do not work, much will be learned that lead to new ideas for more accurate measurements in future trials. This means that clinicians can attempts to pilot new methods, in regular practice helping to accumulate experience and data in support of final validation.
- Need to take risks and adopt a ***creative approach*** to generate potential solutions even if they are not totally viable today but would be the ideal improvement in the field.
- Experts should be brought in who may have ***different perspectives*** on specific ideas or problems to explore the viable solution.
- There is no need to choose one option, but to join efforts to start all ideas and discard the ones that do not work early in the way. A ***fast track communication*** approach to communicate quickly among different stakeholders.
- The use of digital biomarkers and algorithms represents a great opportunity in this field, because from this information could teach about “new” disease features not readable for human eyes or canonical diagnostic and therefore educate to novel endpoints to be measured.
- Worth to explore a ***composite*** that captures a range of common symptoms that might be able to pick up a signal even in a heterogeneous population.
- Very important to ***establish bridges*** between preclinical, clinical researchers and patients to agree on the most important outcomes.
- Collaborate with a range of experts in relevant fields ***to foster a culture of innovation*** and make advances in COAs for more efficient clinical trials.

## Data Availability

Not applicable.
